# Defective Membrane Remodeling in Neuromuscular Diseases: Insights from Animal Models

**DOI:** 10.1371/journal.pgen.1002595

**Published:** 2012-04-05

**Authors:** Belinda S. Cowling, Anne Toussaint, Jean Muller, Jocelyn Laporte

**Affiliations:** 1 Department of Translational Medicine and Neurogenetics, Institut de Génétique et de Biologie Moléculaire et Cellulaire (IGBMC), Illkirch, France; 2 Inserm, U964, Illkirch, France; 3 CNRS, UMR7104, Illkirch, France; 4 Université de Strasbourg, Illkirch, France; 5 Chaire de Génétique Humaine, Collège de France, Illkirch, France; 6 Department of Integrated Structural Biology, Institut de Génétique et de Biologie Moléculaire et Cellulaire, Illkirch, France; 7 Laboratoire de Diagnostic Génétique, CHU Strasbourg Nouvel Hôpital Civil, Strasbourg, France; The Jackson Laboratory, United States of America

## Abstract

Proteins involved in membrane remodeling play an essential role in a plethora of cell functions including endocytosis and intracellular transport. Defects in several of them lead to human diseases. Myotubularins, amphiphysins, and dynamins are all proteins implicated in membrane trafficking and/or remodeling. Mutations in myotubularin, amphiphysin 2 (BIN1), and dynamin 2 lead to different forms of centronuclear myopathy, while mutations in myotubularin-related proteins cause Charcot-Marie-Tooth neuropathies. In addition to centronuclear myopathy, dynamin 2 is also mutated in a dominant form of Charcot-Marie-Tooth neuropathy. While several proteins from these different families are implicated in similar diseases, mutations in close homologues or in the same protein in the case of dynamin 2 lead to diseases affecting different tissues. This suggests (1) a common molecular pathway underlying these different neuromuscular diseases, and (2) tissue-specific regulation of these proteins. This review discusses the pathophysiology of the related neuromuscular diseases on the basis of animal models developed for proteins of the myotubularin, amphiphysin, and dynamin families. A better understanding of the common mechanisms between these neuromuscular disorders will lead to more specific health care and therapeutic approaches.

## Introduction

Membrane remodeling occurs in diverse and essential cellular processes, including endocytosis, intracellular transport, and synaptic vesicle fusion. There are numerous proteins related to membrane remodeling that have diverse functions, including regulation of lipids, membrane adaptor proteins, or cytoskeletal organization. Several genes implicated in membrane remodeling and trafficking are mutated in different forms of human neuropathies (*DNM2*, *MTMR2*, *MTMR13*, *NEFL*, *RAB7A*, *FGD4*, *FIG4*, *SH3TC2*, LITAF/SIMPLE) and myopathies (*MTM1*, *BIN1*, *DNM2*, *CAV3*, *DYSF*) (reviewed in [1]).

The purpose of this review is to discuss how several protein families with functions in membrane remodeling act in the same pathway and how defects of several of these proteins can induce similar human diseases. We consider here animal models of the myotubularin/amphiphysin/dynamin pathway, highlighted by their common implication in both centronuclear myopathies (CNM) and peripheral Charcot-Marie-Tooth neuropathies (CMT). Myotubularins are phosphoinositides phosphatases, amphiphysins are BAR (BIN1, Amphiphysin and RVS167) proteins sensing membrane curvature and regulating membrane remodeling, and dynamins are large GTPases able to tubulate and eventually cleave membranes. Members of these protein families are commonly mutated in several neuromuscular diseases affecting different tissues, suggesting a common pathological pathway and tissue-specific regulations. As other neuromuscular disease genes encode for proteins implicated in membrane transport, this proposed pathological pathway may link together a larger number of membrane trafficking proteins.

## The Myotubularin Family

**Figure 1:**
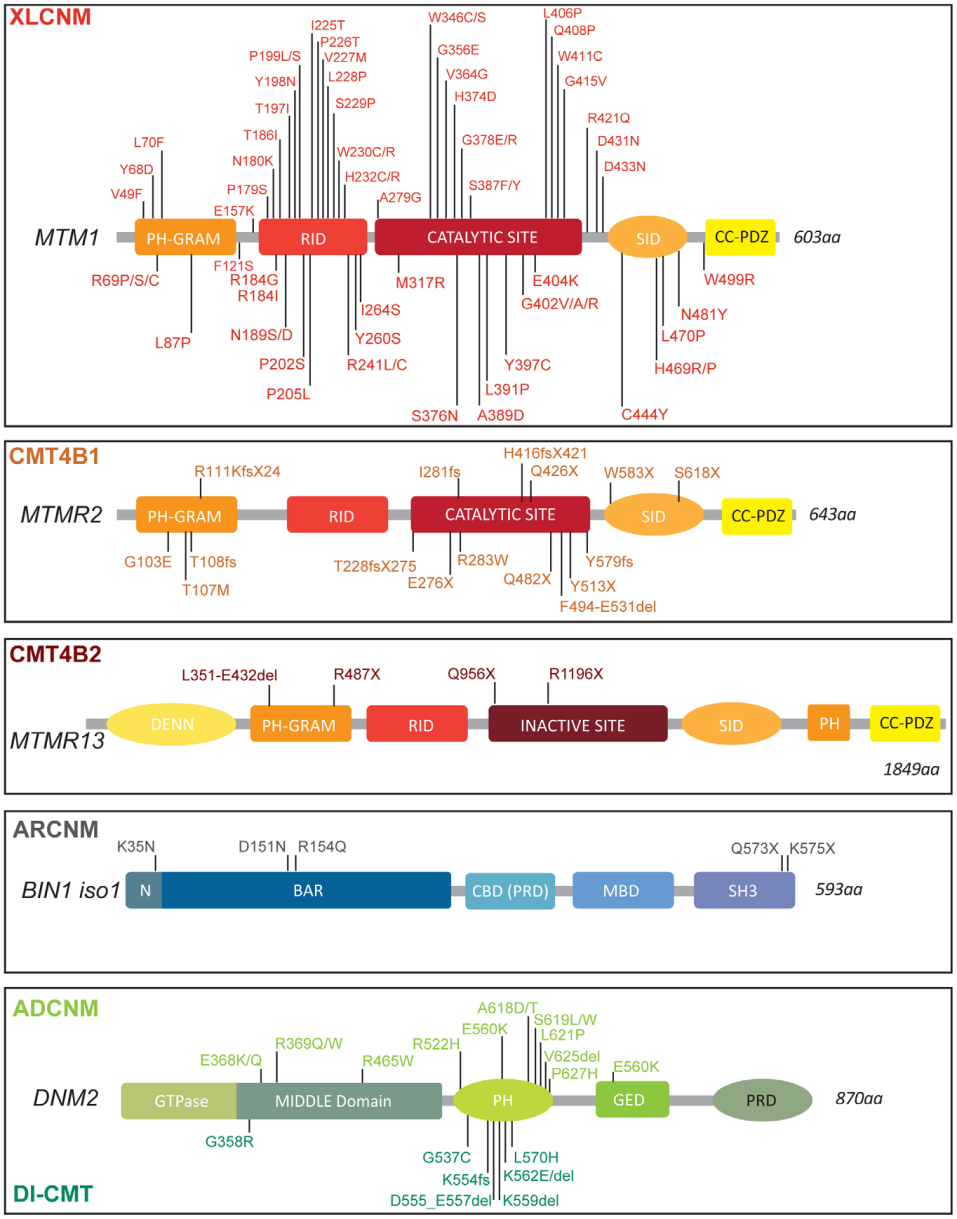
Protein domains and disease-causing mutations in the myotubularin, amphiphysin, and dynamin families. Myotubularin contains a PH-GRAM domain that may bind lipids and a coil-coiled-PDZ binding site to form homo- and hetero-dimers with other members of the myotubularin family. Only the disease-causing missense mutations in MTM1 are represented, based on the international UMD-MTM1 database, existing currently in a local version in Strasbourg (France). MTM1 mutations identified in more than two patients are R69C(9 families), P205L(5), V227M(3), R241C(13), G378R(4), E404K(4), and Y397C(5). AMPH1 and BIN1 possess an N-BAR domain able to sense and eventually curve membrane and a C-terminal SH3 domain binding to proteins with proline-rich domains, such as dynamins [48], [88]. In addition some isoforms have clathrin-binding and Myc-binding domains (CBD, MBD); a phosphoinositide-binding motif is present between the BAR and MBD domains specifically in skeletal muscle. DNM2 contains a GTPase domain, a central middle (MID) domain, a Pleckstrin Homology (PH) domain, a GTPase Effector Domain (GED), and a C-terminal Proline Rich Domain (PRD). Dominant mutations in DNM2 lead to either centronuclear myopathy (above), or peripheral CMT neuropathy (below). Only coding mutations are listed for all genes.

Myotubularin proteins are mutated in two human diseases, centronuclear myopathy (XLCNM, OMIM 310400) and CMT neuropathy. *MTM1* is mutated in the X-linked, most severe form of CNM [2], [3]. Boys with *MTM1* mutations causing X-linked CNM present a very severe and generalized muscle weakness at birth (Figure 1). Death normally occurs within the first year of life due to respiratory failure. Centralized nuclei in hypotrophic fibers are a prominent feature in muscle [2], [3].

CMT comprises a genetically heterogeneous group of inherited disorders affecting myelinated axons in the peripheral nervous system. The disease is characterized by progressive distally accentuated muscle weakness and atrophy. CMT has been subdivided into demyelinating, axonal and intermediate forms on the basis of clinical, electrophysiological, and histological data. CMT4B are severe demyelinating autosomal recessive inherited neuropathies. They are divided in two subgroups (Figure 1; Table S1), CMT4B1 (*MTMR2* mutations, OMIM 601382) and CMT4B2 (*MTMR13* mutations, OMIM 604563) [4]–[7].

Myotubularin (MTM1) is the founding member of a large family of phosphoinositide phosphatases (Figure 1). Myotubularins are 3-phosphatases that play an essential role in maintenance of the spatial and temporal equilibrium of phosphoinositides (PIs), molecular membrane flags that have key roles in membrane identity and protein recruitment [8], [9]. Via its tyrosine phosphatase-like (PTP) domain, MTM1 dephosphorylates phosphatidylinositol 3-phosphate (PtdIns3*P*) and PtdIns(3,5)*P*_2_, second messengers produced by PI 3-kinase (PI3K) and 5-kinase (PI5K) respectively, with important roles in endocytosis and membrane trafficking [10].

**Figure 2:**
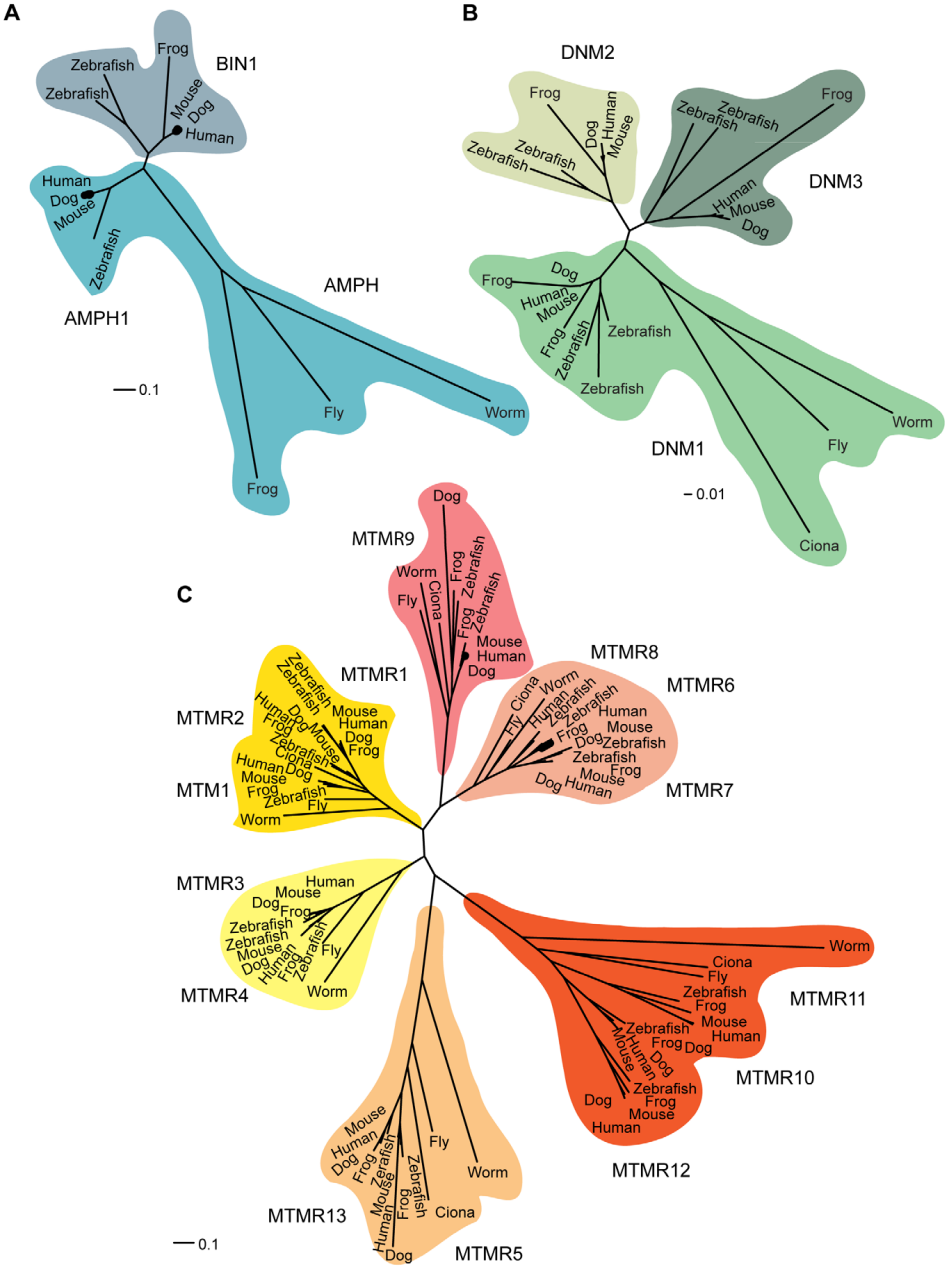
Phylogenetic relationships. Phylogenetic relationships within the amphiphysin (A), dynamin (B), and myotubularin (C) protein families. Sequences were collected using the eggNOG database, which groups genes into families at different taxonomic levels. A high quality multiple sequence alignment was computed for each protein family on all proteins members including, respectively, 91 myotubularin protein sequences, 23 dynamin protein sequences, and 13 amphiphysin protein sequences. For a more detailed description, see Protocol S1. Scale represents the percentage of divergence.

In mammals, the myotubularin family is composed of MTM1 and 13 MTM1-related (MTMR) proteins named MTMR1 to MTMR13 [11], [12] (Figure 2). All contain PTP-like and PH-GRAM domains. Six MTM family members contain an inactive catalytic site owing to conserved substitutions of several amino acids essential for activity [11], [13], [14]. These dead phosphatases, MTMR5 and MTMR9–13, heterodimerize with active myotubularins to regulate their phosphatase activity [12]. To date five pairs of active-dead phosphatases have been confirmed, MTM1-MTMR12, MTMR2-MTMR5, MTMR2-MTMR13, MTMR7-MTMR9, and MTMR6-MTMR9 [15]–[19]. Interestingly mutations in the coupled active and dead phosphatases, MTMR2 and MTMR13, lead to very similar neuropathies, distinguished only by the age of onset and the major involvement of proximal muscles in CMT4B1, often resulting in a more severe neuropathy.

### Myotubularin *Drosophila* Model

How the balance between specific kinases and phosphatases that regulate PI levels, and how disruption of this coregulation may lead to neuromuscular diseases remains unresolved. Studies using *Drosophila* have shown that *Drosophila* myotubularin (*mtm*, the ortholog of human *MTM1* and *MTMR2*) coordinates with class II PI3K to modulate levels of PtdIns3*P* within the cell, which is important in regulating endolysosomal functions and cortical actin remodeling [20], and plays a role in integrin-mediated attachment of myofibers [21]. Integrin accumulated with PtdIns3*P* on endosomal vesicles when mtm was depleted, and integrin localization defects have also been observed in CNM patients [21]. This suggests that *mtm*/PI3K regulation may coordinate integrin trafficking and recycling to the plasma membrane. Modulating class II PI3K levels may therefore constitute a potential therapeutic target for CNM.

### Myotubularin *Caenorhabditis elegans* Model

In *C. elegans*, six myotubularins have been identified, including three active phosphatases MTM-1, MTM-3, and MTM-6, and three dead phosphatases, MTM-5, MTM-9, and MTM-10 [11], [22]–[24], suggesting that the cooperation between active/dead phosphatases is conserved through evolution [17], [25], [26]. Specifically the MTM-6/MTM-9 complex regulated endosomal trafficking of the *Wnt* signaling complex [27]. However all myotubularins appear to be required for the endocytosis of fluid in coelomocytes from the pseudocoelome, a process called coelomocyte uptake (Table S1) [24], [26], [28]. Both *mtm-1* and *mtm-6* play a role in coelomocytes endocytosis, most likely by antagonizing *Let-512* function (a PI3K in *C. elegans*, and a *Vps34* homolog) [24]. A balance between *mtm-1* and *Vps-34* also regulates cell corpse engulfment of apoptotic cells, as MTM-1 inhibited this process via its phosphatase activity [28], [29]. In both mechanisms, MTM-1 regulation of PtdIns3*P* levels could be essential for membrane remodeling. Human MTM1 can substitute the worm MTM-1 function in cell corpse engulfment suggesting that MTM1, or its closer homologue MTMR2, can act similarly in human peripheral nerve or skeletal muscle, two tissues in which defects in MTM1 or MTMR2 induce diseases.

### Myotubularin-Defective Animal Models for Muscle Pathologies

#### mtm-1 zebrafish morphants

Dowling et al. have generated a zebrafish model for X-linked CNM by morpholino knockdown of zebrafish MTM1 [30], which exhibited a delayed chorion hatching and a diminished touch-evoked escape behavior. Abnormally located nuclei, hypotrophy and organelle disruption, and neuromuscular junction disorganization were observed, as seen in patient muscle biopsies [30], [31]. The role of MTM1 as the primary PtdIns3*P* phosphatase in skeletal muscle development was reinforced by an increase of PtdIns3*P* level observed in the skeletal muscle of myotubularin morphants, but not in whole embryos. The rescue of the early muscle weakness by MTMR1 and MTMR2, the closest MTM1 paralogs, suggested that the specific muscle defect induced by a loss of MTM1 is due to the lack of compensation by other PtdIns3*P* myotubularin phosphatases in muscle [30]. Reduction of myotubularin in skeletal muscle affected the organization and the morphology of the T-tubules and sarcoplasmic reticulum (SR) and suggested that the regulation of PtdIns3*P* and PtdIns(3,5)*P*_2_, is essential for the excitation-contraction coupling machinery (Table S1). Indeed, impairment of MTMR14/JUMPY, a phosphatase sharing similar enzymatic activity to MTM1, led to T-tubule anomalies and PtdIns(3,5)*P*_2_ dependent defect of excitation-contraction coupling in mouse skeletal muscles [32], [33].

#### MTM1 mammalian models

The murine model of X-linked CNM generated by targeted mutagenesis of MTM1 [34] exhibited a progressive motor deficit and amyotrophy. Contrary to patients who display a strong muscle weakness from birth, knockout (KO) muscles appeared normal up to 2 wk of age. Mice then exhibited a progressive decrease in muscular strength with similar histopathology to patients [34], [35]. Death occurred around 7–12 wk probably because of cachexia and respiratory insufficiency. Tissue-specific excision of *Mtm1* exon 4 confirmed that the muscle phenotype is due to loss of MTM1 function in skeletal muscle and not in nerves [34]. Altogether, this suggests that MTM1 is not essential for myogenesis but is important for muscle structural maintenance (Table 1). As in zebrafish myotubularin morphants, the loss of myotubularin in mouse skeletal muscle induced abnormal organization of the triads and T-tubules, which appeared prior to defects in excitation-contraction coupling [30], [35]. Similar triad defects have been noted in patients with mutations in MTM1 [36] and in Labrador retrievers with a mutation in MTM1, the latter providing a large animal model in which therapeutic trials could be envisaged [37]. MTM1 was shown to directly bind and regulate desmin localization, and MTM1 mutations causing CNM result in abnormal intermediate filament assembly and architecture, and perturbed mitrochondrial dynamics [38]. Re-expression of *Mtm1* cDNA was sufficient to ameliorate the established muscle weakness in *Mtm1* knockout mice and rescued desmin aggregation [38], [39]. Altogether, myotubularin might specifically regulate the appropriate organization and/or maintenance of triads through fine regulation of specific PI pools at the plasma and reticulum membranes, in skeletal muscle. These results provided new insight into the mechanisms causing CNM and indicated a common pathological link between CNM and desmin-related myopathies.

**Table 1:** Myotubularin/amphiphysin/dynamin protein functions in specific tissues, based on animal and cell models.

Gene	Mouse Model(s)	Specific Muscle Function(s)	Specific Nervous System Function(s)	Reference(s)
*Mtm1*	KO (CMV-Cre) is postnatally lethal week 6–14, most likely due to cachexia and respiratory insufficiency	Essential for muscle maintenance; organization and morphology of T-tubules/sarcoplasmic reticulum (SR)/mitochondria; nuclei position and fiber type expression; integrin localization and PIs important for muscle cells attachment		[21], [30], [34], [35]
	Muscle KO (HSA-Cre) similar viability to total KO			
	Neuronal KO (NSE-Cre) viable and no obvious symptoms (up to 14 wk)			
*Mtmr2*	KO mice survive ≥15 mo of age, progressive CMT4B1-like neuropathy		Myelin sheath maintenance	[6], [41], [42], [89]
	Schwann cell KO mice survive ≥15 mo; dysmyelinating phenotype similar to total KO			
	Motor neuron KO mice survive ≥12 mo, no dysmyelination or axonopathy observed			
*Mtmr13*	KO mice indistinguishable to wildtype until ≥15 mo of age, exhibit progressive CMT4B2-like neuropathy		Myelin sheath maintenance	[19], [43]
*Amph1*	Premature death due to spontaneous seizures; 50% survive to 10 mo old		Synaptic vesicles recycling in brain	[56]
*Bin1*	KO is perinatally lethal (often within 24 h of birth), exhibit cardiac structural defects	Membrane remodeling at T-tubules?		[55]
*Dnm1*	KO is postnatally lethal, within 2 wk of birth, probably due to synaptic vesicle endocytosis defects		Essential role in neurotransmission and synaptic vesicle endocytosis during intense stimulation	[73], [77], [79]
*Dnm2*	KO is embryonically lethal (prior to embryonic day 10)	Important for muscle maintenance; maintenance of T-tubule, reticulum, and mitochondrial network		[67], [80]–[82]
	R465W HMZ KI is perinatally lethal within 24 h of birth, may be due to clathrin-mediated endocytosis defects			
	R465W HTZ KI mice are viable with no reduced lifespan, myopathic phenotype			

### Myotubularin-Related Animal Models for Neuropathies

#### MTMR2 and MTMR13 murine models

*Mtmr2*-deficient mice start developing peripheral nerve abnormalities at 1 mo of age (Table 1) [40]–[42]. They present a dysmyelinating phenotype similar to CMT4B1 patients, consisting of motor and peripheral neuropathy with myelin outfoldings and redundant loops appearing as membrane extensions originating predominantly from paranodes, a region in between compact myelin and nodes of Ranvier. Expression of the MTMR2 binding protein Dlg-1/SAP97, a scaffolding protein belonging to the MAGUK protein family, decreased in *Mtmr2*-null mice, suggesting that loss of interaction between MTMR2 and Dlg-1/SAP97 disrupts cell polarity and cellular junctions or membrane remodeling in Schwann cells, leading to membrane outfoldings at paranodes. Two *Mtmr13*-deficient mouse models have been generated, both of which led to a loss of MTMR13 expression and reproduced the CMT4B2 phenotype, including myelin outfoldings and infoldings (Table 1) [43], [44]. As in *Mtmr2*-deficient mice, myelin outfoldings were found near nodes of Ranvier and increased in number and complexity with age [43], [44]. Double *Mtmr2*/*Mtmr13*-deficient mice presented no additional or more severe phenotype, suggesting MTMR13 regulates MTMR2 [43] and is consistent with data showing a strong increase of MTMR2 phosphatase activity when MTMR2 and MTMR13 formed a complex [19], [45].

## The Amphiphysin Family

The autosomal recessive form of CNM (ARCNM, OMIM 255200) normally presents with muscle weakness typically appearing in infancy or early childhood, although cases were reported to present strong hypotonia at birth [46], [47]. The mutated gene associated with some cases of ARCNM is *BIN1*, encoding amphiphysin 2 (Figure 1) [46]. Amphiphysins belong to the BAR protein family [48]. They play roles in membrane remodeling and are conserved between humans and *Drosophila*, with functional orthologs found in yeast. In mammals, two genes encode for amphiphysins; amphiphysin1 (*Amph1*) and amphiphysin2 (*BIN1*) (Figure 2). *Amph1* is expressed in brain and is implicated in synaptic endocytosis. BIN1, initially described as a tumor suppressor via its c-myc binding domain, is ubiquitously expressed although different isoforms are formed by complex alternative splicing that appears tissue specific [36], [49]. The muscle-specific isoform of BIN1 (iso8) contains a phosphoinositides-binding domain, and plays a role in T-tubule biogenesis [50], [51]. Nevertheless, mutations leading to CNM are found in common domains of the numerous BIN1 isoforms. Auto-antibodies against AMPH1 lead to a rare auto-immune disease called Stiff-man syndrome, involving chronic muscle rigidity and episodic spasms, sometimes with a paraneoplastic origin [52].

### Amphiphysin *Drosophila* Model

The fly model has been generated by disruption of the only gene encoding amphiphysin (*Amph*) in *Drosophila* [53]. Adult *Amph*-deficient flies displayed no synaptic vesicle defects but were flightless and sluggish. Loss of *Amph* resulted in severe disorganization and reduction of the T-tubules, similar to the triads defect observed in CNM patients with BIN1 mutations [36] and in *Mtm1*-deficient mice and *mtm1*-deficient Zebrafish, reinforcing the hypothesis that MTM1 and amphiphysin 2 work in the same pathway in skeletal muscle.

### Amphiphysin *C. elegans* Model

AMPH-1 is the only amphiphysin in *C. elegans*. AMPH-1 directly interacts and colocalizes with the membrane tubulating ATPase RME-1 (EHD ortholog) on membrane tubules from recycling endosomes [54]. Similarly to RME-1 mutants, loss of AMPH-1 induced a defect in basolateral recycling in the polarized intestinal epithelium. AMPH-1 may function in part to recruit RME to membrane of recycling endosomes [54]. All together these data suggest that AMPH-1 is implicated in recycling, probably through membrane remodeling and RME-1 recruitment (Table S1).

### Amphiphysin 2/BIN1 Mouse Models

Deletion of the exon 3 in *Bin1* led to loss of protein in homozygous mice [55]. *Bin1*-null embryos developed normally but died perinatally, potentially because of occlusion of the ventricular chambers. Unexpectedly there was no demonstrable impact of BIN1 loss on apoptosis, actin cytoskeletal organization, endocytosis, or on the specialized phagocytic endocytosis. Cardiac myofibrils were loosely packed and disorganized, with defective sarcomere units, diffuse Z-lines, and no apparent A-bands. Skeletal muscle was not thoroughly analyzed [55]. Moreover, as in *Drosophila*, *Bin1*-deficient mice presented normal brain architecture and synaptic vesicles, reinforcing the non-essential function of BIN1 in brain.

### Amphiphysin 1 Murine Model

*Amph1*-deficient mice have been created by deletion of exon 1 (Table 1) [56]. AMPH1 and BIN1 form heterodimers in the brain, and absence of AMPH1 resulted in a strong reduction in BIN1 level in brain but not skeletal muscle [56]. Conversely, the expression level of *Amph1* was not altered in the brain of *Bin1*-deficient mice, suggesting that *Bin1* is not essential for *Amph1* stability and function in brain [55]. *Amph1*-deficient mice suffered severe uncontrollable seizures, leading to reduced viability with 50% of mice dying by 40 wk of age. A reduction in synaptic vesicle recycling efficiency and reduced recruitment of AP-2 (alpha adaptin) and clathrin to membranes were detected [56]. AMPH1 is localized to the tubulobulbar complex (TBC) in Sertoli cells, and absence of AMPH1 led to a reduced number of plasma membrane invaginations and a subsequent increase number of unreleased spermatids [57].

According to the different animal models generated for amphiphysins and other cell studies, AMPH1 and BIN1 appear to have specialized functions depending on their splice isoforms and tissues where they are expressed. AMPH1 and brain isoforms of BIN1 might be dedicated to endocytosis and/or recycling, and non-neuronal AMPH1 and BIN1 proteins play a major role in plasma membrane remodeling and trafficking (Tables 1 and S1).

## The Dynamin Family

Although ubiquitous expression and the central role of DNM2 in endocytosis suggested an essential function in all tissues, *DNM2* was found mutated in two tissue-specific human diseases: autosomal dominant centronuclear myopathy (ADCNM, OMIM 160150) and dominant intermediate Charcot-Marie-Tooth neuropathy (DI-CMTB, OMIM 606482) (Figure 1) [58], [59]. The autosomal dominant form of CNM is generally clinically mild and usually appears in adults with a diffuse weakness that is slowly progressive and may be accompanied by muscle hypertrophy. However the severity varies from mild late-onset to severe neonatal onset [58], [60]–[63]. *DNM2* mutations are also linked to either DI-CMTB with intermediate conduction velocity and associated neutropenia or axonal CMT. How different mutations in the same gene cause two diseases affecting different tissues (peripheral nerve and muscles) remains unexplained.

Dynamins belong to a large family of GTPases involved in formation and fission of budding vesicles from the plasma membrane and Golgi [64], [65]. In humans, the dynamin family comprises DNM1, DNM2, and DNM3 (Figure 2). DNM2 is ubiquitously expressed, DNM1 is present mainly in brain, and DNM3 is detected in brain and testis. Specific isoforms of each dynamin have been linked to specific subcellular localization and functions [66], [67]. Via the Pleckstrin homology (PH) and proline rich domains (PRD) domains, DNM2 binds respectively to the phosphoinositides PtdIns(4,5)*P*_2_, and to Src-Homology-3 (SH3) domain proteins including amphiphysins.

### *Drosophila* Dynamin Models

In *Drosophila* the *shibire (shi)* gene encodes for the ortholog of mammalian dynamins. At non-permissive temperatures, the *shi* ^ts1^ and *shi* ^ts2^ mutations prevent GTP hydrolysis and *shibire* flies display disrupted synaptic vesicle endocytosis and depleted synaptic terminal vesicles [68]–[70]. Synaptic vesicles were trapped at a “collared-pit” stage in membrane internalization, and inactivation of the GTPase domain abolished vesicle translocation [70]. Dynamin regulates endocytosis in a variety of tissues [69], [71]. In cardiac tissue, recessive *shi* ^ts1^ and *shi* ^ts2^ mutations exhibited bradychardia and arrhythmia because of defects in electrical communication throughout the myocardium [72].

### Dynamin *C. elegans* Models

Dyn-1 is the unique dynamin expressed in *C. elegans*. *Dyn-1(ky51)* mutant worms had uncoordinated movement, kinked posture, and were sluggish at non-permissive temperatures [73]. This is consistent with the enrichment of Dyn-1 in motor neurons that innervate bodywall muscles and head musculature, required respectively for locomotion and head movement [73]. Dyn-1 concentration in the nerve ring indicated a role in synaptic vesicle recycling [74]. In non-neuronal cells, *Dyn-1* defects in coelomocytes led to decreased fluid-phase endocytosis. *Dyn-1 (n4039)* worms revealed that dynamin also has a role in engulfment and degradation of apoptotic cells during embryogenesis [75]. Interestingly, human DNM2 could perform a similar function to Dyn-1 in *C. elegans* [75]. These results suggest that dynamin proteins can have similar functions when expressed in the same tissue. In gonadal sheath cells, absence of Dyn-1 led to accumulation of enlarged vesicles, vesicles interconnected via lipid tubules, and plasma membrane-connected vesicles, all suggesting defects in fission [75]. A role for Dyn-1 in membrane transport has also been characterized for the maintenance of anterior polarity in *C. elegans* embryos (Table S1) [76].

### Dynamin 1 Mammalian Models

*Dnm1*-deficient mice were indistinguishable from littermates at birth, however decreased ingestion of milk and poor motor coordination were detected, and mice usually died within 2 wk [77]. Analyses of the structure and function of synapses from *Dnm1*-deficient mice highlighted that DNM1 was dispensable for endocytic recycling of synaptic vesicles under basal conditions but became essential when intense stimulus imposed a heavy load of endocytosis [77]. Expression of DNM2 or DNM3 rescued partially or efficiently, respectively, the endocytic blockade in DNM1-defective cultured neurons, indicating that all dynamins could participate in synaptic vesicle endocytosis. This was recently confirmed in DNM1/DNM3 double KO [78].

An homozygous Arg256Leu DNM1 mutation was identified in Labrador retrievers affected with exercise-induced collapse [79]. A collapse episode lasts 5–10 min and complete recovery usually occurs after 30 min. Arg256Leu affects an amino acid conserved in DNM2 and DNM3, which is located at the boundary between the GTPase and the MID domains [79]. Similar to *Drosophila* and *C. elegans* temperature-sensitive phenotypes, the mutation in canine DNM1 may lead to a defect in synaptic vesicle recycling that sustains synaptic transmission during intensive exercise (Table S1).

### Dynamin 2 Murine Models

*Dnm2* KO mice died before embryonic day 10 [80]. In double *Dnm1/Dnm2* null fibroblasts, clathrin-mediated endocytosis is arrested and endocytotic intermediates accumulated in the cytoplasm [80]. Heterozygous R465W-DNM2 knock-in mice, the most common mutation causing ADCNM, exhibited a fairly mild myopathic phenotype, with abnormalities in mitochondrial and reticular networks [81]. However homozygous mice died hours after birth, possibly owing to clathrin-mediated signaling defects, correlating with the above results from *Dnm1/Dnm2* KO cells. A second model was created by intramuscular adeno-associated virus (AAV) injections of R465W-DNM2 into adult wild-type muscle. Mice exhibited muscle weakness associated with disruptions of the mitochondrial and T-tubule networks [82]. This model reproduced most ADCNM features in mice, and was akin to ADCNM patient biopsies [36]. These models provide evidence that ADCNM arises primarily from defects in skeletal muscle rather than peripheral nerves, and that dynamin 2 is crucial in the maintenance of adult muscle fiber structure and function.

## A Common Molecular Mechanism?

**Figure 3:**
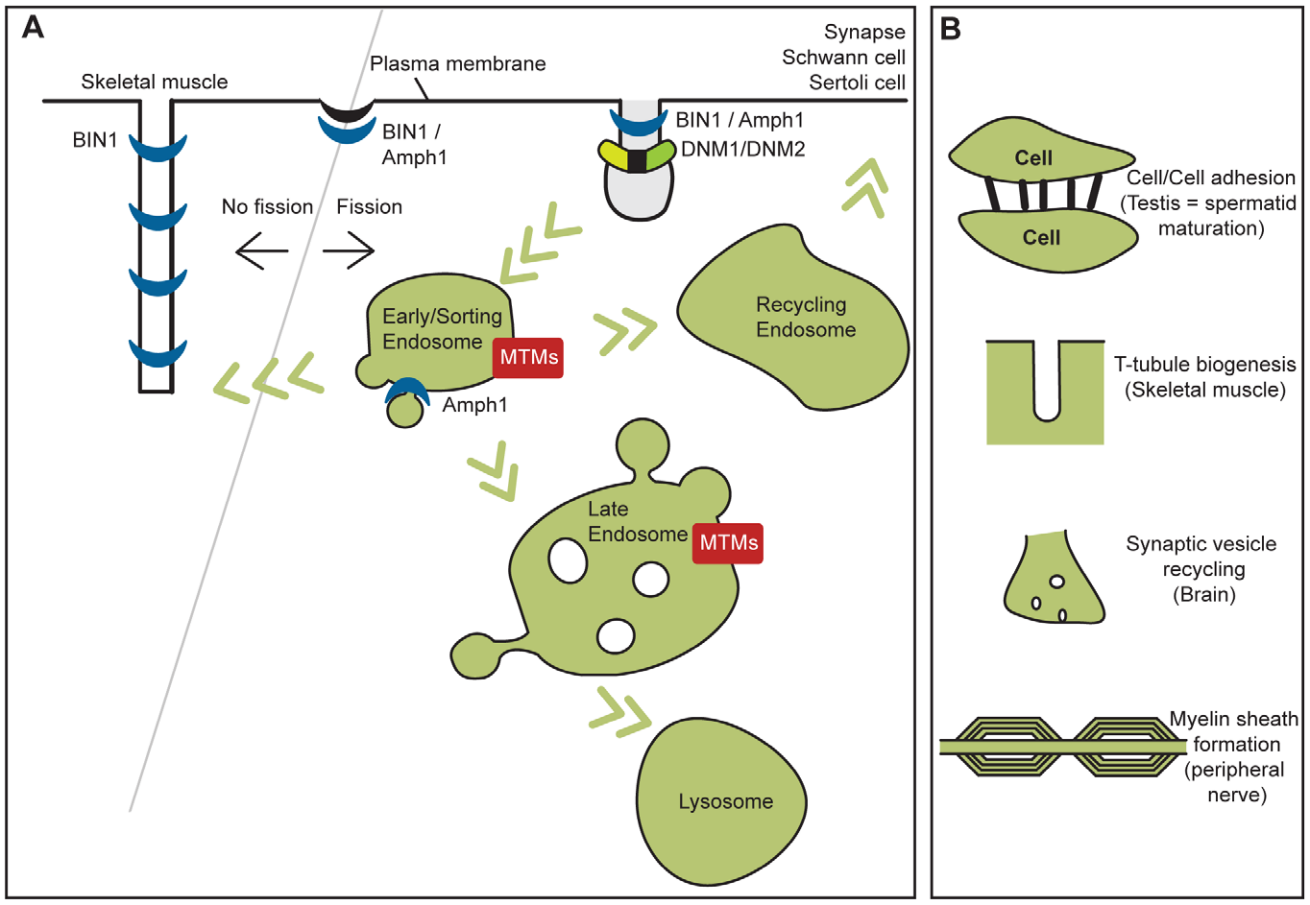
Cellular functions of myotubularins, amphiphysins, and dynamins implicated in human diseases and their related pathological mechanisms. (A) Human diseases, (B) their related pathological mechanisms. Membrane fission is necessary for vesicle formation and subsequent trafficking, while inhibition of membrane fission or membrane addition at the T-tubules in muscle may be necessary for their formation and maintenance.

Myotubularins are phosphatases regulating the level of PIs, amphiphysins can bend membranes, and dynamins oligomerize around the neck of vesicles to promote tubulation and eventually membrane fission. All these proteins fit in a membrane remodeling pathway (Figure 3) and are associated with myopathies and peripheral neuropathies or neurological syndromes. It is not yet clear if mutations causing more severely defective proteins correlate with earlier onset, or more severe forms of the disease, nor is it clear if the location of mutations within genes gives rise to tissue-specific disorders. A more detailed analysis of the impact of mutations on the protein functions is needed to answer these points. The above pathological pathway may also be expanded to include other genes mutated in CMT and other myopathies. This could include several genes mutated in different forms of CMT (e.g., *NEFL*, *RAB7A*, *FGD4*, *FIG4*, *SH3TC2*, LITAF/SIMPLE) (reviewed in [83]) encoding proteins regulating PIs metabolism and membrane trafficking; and myopathies involving mutations in caveolin 3 and dysferlin genes, which also lead to common defects in T-tubules and membrane transport in patients and animal models (Figure 3) [84], . While mutations in several membrane trafficking proteins described here are associated with neuromuscular disorders, in contrast mutations affecting normal lysosomal function induce a separate category of human diseases termed lysosomal storage diseases (LSD), which affect several tissues, most commonly causing progressive neurodegeneration. We propose a hypothesis to explain how different protein families are implicated in similar diseases while, conversely, the same protein families are associated with different neuromuscular diseases (Figure 3; Table 1).

### Neurological Functions

These proteins could coordinate vesicle formation and endocytosis and recycling. Schwann cells require large amounts of plasma membrane to wrap around axons and may be more susceptible than other cell types to membrane transport defects. This pathway is also important at the synapse that requires very rapid trafficking and recycling through membrane fission, transport, and fusion. Amphiphysins and dynamins are well-known interactors and regulators of endocytosis, and dynamins are excellent candidates for membrane fission. Indeed dynamin inhibition leads to decreased separation of vesicles from the plasma membrane [70]. Several myotubularins have been implicated in endocytosis, through regulation of PtdIns3*P* and PtdIns(3,5)*P*_2_ levels in *Drosophila*, *C. elegans*, zebrafish, and mice models, and these PIs were shown to be key players in both endocytosis and exocytosis [8], [9]. Such membrane trafficking is implicated in synaptic vesicle recycling in brain, and in recycling of adhesion molecules that may be important for cell–cell contact and myelin sheath formation or maintenance. Defects in these pathways could explain the hallmarks of neurological syndromes and neuropathies associated with mutations in these protein families.

### Skeletal Muscle Functions

In skeletal muscle, MTM1, BIN1, and DNM2 may coordinate the formation and/or organization of T-tubule/triad structures (Figure 3). Following induction of membrane curvature, the invagination may become elongated to form a T-tubule. BIN1 is highly expressed in skeletal muscle and was implicated in T-tubule biogenesis in *Drosophila* and muscle cells [51], [53], and T-tubule defects are seen in zebrafish and mouse animal models, and in CNM patients with mutations in these three proteins [36]. BIN1 contains a phosphoinositide-binding domain present only in the muscle isoform indicating PIs are involved in this process, however their role and the function of MTM1 in this process remain to be determined. During T-tubule biogenesis and maintenance, additional membrane may be acquired through fusion of endocytic vesicles (similar to the process proposed for Schwann cells in peripheral nerves); defective endocytosis and recycling may therefore contribute to the phenotype observed in patient muscle.

### Tissue-Specific Function

Whilst MTM1 and MTMR2 have very similar sequences (90% identity) and ubiquitous expression, mutations induce two different diseases, highlighting that myotubularin functions are not redundant, and isoforms may perform tissue-specific functions. The membrane remodeling pathway may have different functions in different tissues, and mutations may affect different protein functions. Unlike the myotubularin family, for dynamin 2 the same gene is mutated in two tissue-specific disorders. The tissue-specific function may thus be dictated by the ability of dynamins to promote membrane fission or not. Inhibition or slow GTPase hydrolysis would favor membrane tubules, and fast GTP hydrolysis would lead to fission and vesicle formation. Inhibition of membrane fission will lead to the neurological and neuropathy phenotypes while increased fission may prevent formation of T-tubules and lead to muscle contraction defects. Supporting this theory DNM2 mutations causing CNM have been shown to have increased oligomer stability and GTPase activity [86], [87]. However in contrast to BIN1, DNM2 was not observed at T-tubules to date. Regulation of this balance can be due to tissue-specific isoforms, interactors, or post-translational regulation as most of these proteins are ubiquitous.

## Conclusions and Pending Issues

Animal models were instrumental to decipher the cellular and physiological functions of the implicated proteins and will be an asset to further characterize the underlying pathological mechanisms and test therapeutic approaches. Myotubularins, amphiphysins, and dynamins play a key role in membrane remodeling. Mammalian models could now be used to follow the dynamics of membrane remodeling and trafficking in the affected tissues and to characterize the tissue-specific regulation of the membrane fission process. Phosphoinositides associated with these proteins are involved in membrane trafficking; however, the molecular link between phosphoinositides and membrane remodeling and how this relates to T-tubule formation and myelin maintenance remain to be deciphered.

A glossary and useful links can be found in Text S1.

## Supporting Information

Protocol S1 Supplementary bioinformatics methods.
(DOC)

Table S1 Animal models for the myotubularin/amphiphysin/dynamin families and linked human diseases.
(DOC)

Text S1 Glossary and useful links.
(DOC)
