# Supplementary material for: Defective Membrane Remodeling in Neuromuscular Diseases: Insights from Animal Models
Source: PLoS Genet. 2012 Apr 5;8(4):e1002595. doi: 10.1371/journal.pgen.1002595 (PMC3320571; doi:10.1371/journal.pgen.1002595)
Supplement: Protocol S1 — Supplementary bioinformatics methods. (DOC) [file pgen.1002595.s001.doc]

**Supplementary bioinformatics methods**

## The sequences of the three proteins families (Myotubularin, Dynamin and Amphiphysin) were collected using the version 2 of the eggNOG database [1] which groups genes into families at different taxonomic levels balancing phylogenetic coverage and resolution. The metazoan Non Supervised Orthologous Groups (meNOG) level was chosen as a reference for each of the three protein families. We used the following groups: meNOG04077 (DNM1, DNM2 and DNM3), meNOG07640 (BIN1 and BIN2), meNOG05427 (MTM, MTMR1 and MTMR2), meNOG05852 (MTMR3 and MTMR4), meNOG04791 (MTMR6, MTMR7 and MTMR8), meNOG14262 and meNOG05509 (MTMR5 and MTMR13), meNOG04913 (MTMR5 and MTMR13), meNOG06614 (MTMR10), meNOG12975 (MTMR11) and meNOG10800 (MTMR12). For the purpose of building a complete Multiple Sequence Alignment (MSA) for the Myotubularin family the concerned meNOGs were fused.

The list of 34 metazoan species covered by the meNOG was reduced to encompass the following major model organisms (taxonomic identifiers are given into parenthesis): *Caenorhabditis elegans* (6239)*, Drosophila melanogaster* (7227)*, Ciona intestinalis* (7719), *Danio rerio* (7955)*, Xenopus tropicalis* (8364)*, Canis lupus familiaris* (9615)*, Mus musculus* (10090) and *Homo sapiens* (9606).

A high quality MSA of all proteins from each of these datasets was then computed using AQUA [2]. The MSA was manually refined, taking into account secondary structures and resulting in a final MSA including respectively 91 myotubularin proteins sequences, 23 dynamin protein sequences and 13 amphiphysin protein sequences. The complete MSAs are available upon request.

Gblocks [3] was then used on each MSA to remove the badly aligned regions (using the default settings, except for the following: Minimum Number Of Sequences For A Flanking Position = Half of the total number of sequences in the MSA, Minimum Length Of A Block = 2; Allowed Gap Positions = all). This step resulted in the selection of 367 amino acid position (out of 3112) for the myotubularin MSA, 432 amino acid positions (out of 1048) for the amphiphysin MSA, and 860 amino acid positions (out of 1052) for dynamin MSA. Following this, PhyML [4] was used to find the maximum likelihood tree for each families. The parameters used were as follows: 100 bootstrap replicates, the JTT model of evolution, with the proportional of invariable sites estimated. Site rate-heterogeneity was estimated using a gamma model with an estimated alpha parameter. Rate heterogeneity was summarized using 4 categories of sites.

In general, sequence format conversion (e.g. MSA in fasta format to phylip format) to allow crosstalk between those programs was done using the ReadSeq program [5]. The constructed phylogenetic hypotheses were visualized with iTOL [6] using the unrooted mode. The phylogenetic trees are available using the following URLs:

Amphiphysin:

<http://itol.embl.de/external.cgi?mode=unrooted&tree=1307979811189812816980920>

Myotubularin : <http://itol.embl.de/external.cgi?mode=unrooted&tree=1307979811166012816972680>

Dynamin :

<http://itol.embl.de/external.cgi?mode=unrooted&tree=1307979811184212816979740>

**References**

1. Muller J, Szklarczyk D, Julien P, Letunic I, Roth A, et al. (2010) eggNOG v2.0: extending the evolutionary genealogy of genes with enhanced non-supervised orthologous groups, species and functional annotations. Nucleic Acids Res 38: D190-195.

2. Muller J, Creevey CJ, Thompson JD, Arendt D, Bork P (2010) AQUA: automated quality improvement for multiple sequence alignments. Bioinformatics 26: 263-265.

3. Talavera G, Castresana J (2007) Improvement of phylogenies after removing divergent and ambiguously aligned blocks from protein sequence alignments. Syst Biol 56: 564-577.

4. Guindon S, Gascuel O (2003) A simple, fast, and accurate algorithm to estimate large phylogenies by maximum likelihood. Syst Biol 52: 696-704.

5. Gilbert D (2003) Sequence file format conversion with command-line readseq. Curr Protoc Bioinformatics Appendix 1: Appendix 1E.

6. Letunic I, Bork P (2007) Interactive Tree Of Life (iTOL): an online tool for phylogenetic tree display and annotation. Bioinformatics 23: 127-128.
