# Supplementary material for: Defective Membrane Remodeling in Neuromuscular Diseases: Insights from Animal Models
Source: PLoS Genet. 2012 Apr 5;8(4):e1002595. doi: 10.1371/journal.pgen.1002595 (PMC3320571; doi:10.1371/journal.pgen.1002595)
Supplement: Table S1 — Animal models for the myotubularin/amphiphysin/dynamin families and linked human diseases. (DOC) [file pgen.1002595.s002.doc]

**Table S1. Animal models for the myotubularin/amphiphysin/dynamin families, and linked human diseases.**

| Gene | Chromo-some | Protein | Species | Human disease or animal model phenotype | Ref(s) |
| --- | --- | --- | --- | --- | --- |
| MYOTUBULARINS FAMILY | | | | |  |
| *MTM1* | Xq28 | MTM1/ myotubularin | Human  (*Homo Sapiens*) | XLCNM: Neonatal hypotonia and muscle weakness. Centralized nuclei, muscle fiber hypotrophy, type I predominance. | [1] |
| *MTM1* | X | MTM1 | Dog  (Canis Familiaris) | Spontaneous mutation (N155K): Progressive generalized muscle weakness. Atrophic fibres with centralized nuclei, necklace fibres, mitochondrial and T-tubule abnormalities. | [2] |
| *Mtm1* | X 27.8 cM | MTM1 | Mouse  (*Mus Musculus*) | KO: Muscle weakness, atrophic myofibres with central nuclei, type I predominance, T-tubule abnormalities. | [3,4] |
| *mtm1* | 7 | mtm-1 | Zebrafish  (*Danio Rerio*) | Muscle weakness, atrophic myofibres with central nuclei, predominance of type I, T-tubules abnormalities. | [5] |
| *MTMR2* | 11q22 | MTMR2 | Human  (*Homo Sapiens*) | CMT4B1: Progressive dysmyelination of peripheral nerve. Muscle weakness and atrophy. | [6] |
| *Mtmr2* | 9 | MTMR2 | Mouse  (*Mus Musculus*) | KO/KI: Motor and peripheral neuropathy, myelin oufoldings, infoldings and redundant loops. | [7,8] |
| *mtm* | 2L | myotubularin | Fly  (*D. Melanogaster*) | RNAi: Endolysosomal and cortical actin remodeling defects. Mutants: Integrin localization defect. | [9,10] |
| *MTMR13* | 11p15.4. | MTMR13/ SBF2 | Human  (*Homo Sapiens*) | CMT4B2: Progressive dysmyelination of peripheral nerve. | [11,12] |
| *Mtmr13* | 7 F2 | MTMR13 | Mouse  (*Mus Musculus*) | KO: Motor and peripheral neuropathy, myelin outfoldings, infoldings and redundant loops. | [13,14] |
| *Mtmr5* | 15 F1 | MTMR5/ SBF1 | Mouse  (*Mus Musculus*) | KO: Male infertility, spermatogenesis deficiency. | [15] |
| *mtm-1* | I | mtm-1 | Worm  (*C. elegans*) | mtm1 RNAi: Coelomocytes uptake defects, cell corpse engulfment increased. | [16,17] |
| *mtm-3* | III | mtm-3 | Worm  (*C. elegans*) | RNAi : Egg laying defect. | [18] |
| *mtm-6* | III | mtm-6 | Worm  (*C. elegans*) | RNAi: Coelomocytes uptake defect. Mutant: defects in Wnt-dependent processes. | [18,19] |
| *mtm-9* | V | mtm-9 | Worm  (*C. elegans*) | RNAi: Coelomocytes uptake defect. Mutant: defects in Wnt-dependent processes. | [18,19] |
|  |  |  |  |  |  |
| AMPHIPHYSINS FAMILY | | | | |  |
| *BIN1* | 2q14 | BIN1/ amphiphysin 2 | Human  (*Homo Sapiens*) | ARCNM: Progressive muscle weakness, childhood onset, centralized nuclei, muscle fiber hypotrophy. | [20] |
| *Bin1* | 18 14.0 cM | BIN1 | Mouse  (*Mus Musculus*) | KO: Mice lethality, cardiomyopathy. | [21] |
|  |  |  |  | KO in mammary gland: Defect in mammary gland remodeling during pregnancy. | [22] |
| *AMPH1* | 7p14-p13 | AMPH1/ amphiphysin 1 | Human  (*Homo Sapiens*) | Stiff-person Syndrome (due to auto-antibodies): Muscle rigidity that waxes and wanes with concurrent spasms. | [23] |
| *Amph1* | 13 10.0cM | AMPH1 | Mouse  (*Mus Musculus*) | KO: Reduction of synaptic vesicles recycling in brain, increasing propensity of seizure. Learning deficit. Defect in spermatogenesis: reduction of membrane invaginations (tubulobulbar complex) in Sertoli cells. | [24,25] |
| *amph* | 2R | amph | Fly  (*D. Melanogaster*) | KO: Flightless and sluggish, absence or longitudinal organization of T-tubules. | [26] |
| *amph-1* | IV | AMPH-1 | Worm  (*C. elegans*) | Mutant: Defect in recycling endosome in intestinal epithelia. | [27] |
| DYNAMINS FAMILY | | | | |  |
| *DNM2* | 19p13.2 | DNM2/ dynamin 2 | Human  (*Homo Sapiens*) | ADCNM: Progressive muscle weakness, childhood or adult onset, central nuclei in muscle fibres. | [28] |
|  |  |  |  | DI-CMTB: Intermediate form, progressive dysmyelinating peripheral neuropathy. | [29] |
| *Dnm2* | 9 7.0cM | DNM2 | Mouse  (*Mus Musculus*) | KO: Embryonic lethal. R465W KI or AAV transduced: Muscle weakness, atrophy with centralized nuclei, defects in T-tubule and mitochondrial networks. | [30-32] |
| *DNM1* | 9 | DNM1/ dynamin 1 | Dog  (*Canis Familiaris*) | Spontaneous mutation (R256L): Syndrome of exercise-induced collapse, muscle weakness, uncoordination. | [33] |
| *Dnm1* | 2 17.0 cM | DNM1 | Mouse  (*Mus Musculus*) | KO: Defect in endocytosis or/and recycling of synaptic vesicles during intense stimulation. | [34] |
| *Dnm* | X | Dnm | Fly  (*D. Melanogaster*) | Shibire (shi^ts1^ and shi^ts2^) mutants: Defect in membrane remodeling of synaptic vesicle at non permissive temperature. | [35] |
| *dyn-1* | X | Dyn-1 | Worm  (*C. elegans*) | *Dyn1 (k51)* mutant: Defect in synaptic endocytosis in motor neurons and fluid-phase endocytosis in coelomocytes. | [36] |

**References**

1. Laporte J, Hu LJ, Kretz C, Mandel JL, Kioschis P, et al. (1996) A gene mutated in X-linked myotubular myopathy defines a new putative tyrosine phosphatase family conserved in yeast. Nat Genet 13: 175-182.

2. Beggs AH, Bohm J, Snead E, Kozlowski M, Maurer M, et al. (2010) MTM1 mutation associated with X-linked myotubular myopathy in Labrador Retrievers. Proc Natl Acad Sci U S A 107: 14697-14702.

3. Buj-Bello A, Laugel V, Messaddeq N, Zahreddine H, Laporte J, et al. (2002) The lipid phosphatase myotubularin is essential for skeletal muscle maintenance but not for myogenesis in mice. Proc Natl Acad Sci U S A 99: 15060-15065.

4. Al-Qusairi L, Weiss N, Toussaint A, Berbey C, Messaddeq N, et al. (2009) T-tubule disorganization and defective excitation-contraction coupling in muscle fibers lacking myotubularin lipid phosphatase. Proc Natl Acad Sci U S A 106: 18763-18768.

5. Dowling JJ, Vreede AP, Low SE, Gibbs EM, Kuwada JY, et al. (2009) Loss of myotubularin function results in T-tubule disorganization in zebrafish and human myotubular myopathy. PLoS Genet 5: e1000372.

6. Bolino A, Muglia M, Conforti FL, LeGuern E, Salih MA, et al. (2000) Charcot-Marie-Tooth type 4B is caused by mutations in the gene encoding myotubularin-related protein-2. Nat Genet 25: 17-19.

7. Bolino A, Marigo V, Ferrera F, Loader J, Romio L, et al. (2002) Molecular characterization and expression analysis of Mtmr2, mouse homologue of MTMR2, the Myotubularin-related 2 gene, mutated in CMT4B. Gene 283: 17-26.

8. Bonneick S, Boentert M, Berger P, Atanasoski S, Mantei N, et al. (2005) An animal model for Charcot-Marie-Tooth disease type 4B1. Hum Mol Genet 14: 3685-3695.

9. Velichkova M, Juan J, Kadandale P, Jean S, Ribeiro I, et al. (2010) Drosophila Mtm and class II PI3K coregulate a PI(3)P pool with cortical and endolysosomal functions. J Cell Biol 190: 407-425.

10. Ribeiro I, Yuan L, Tanentzapf G, Dowling JJ, Kiger A (2011) Phosphoinositide regulation of integrin trafficking required for muscle attachment and maintenance. PLoS Genet 7: e1001295.

11. Azzedine H, Bolino A, Taieb T, Birouk N, Di Duca M, et al. (2003) Mutations in MTMR13, a New Pseudophosphatase Homologue of MTMR2 and Sbf1, in Two Families with an Autosomal Recessive Demyelinating Form of Charcot-Marie-Tooth Disease Associated with Early-Onset Glaucoma. Am J Hum Genet 72: 1141-1153.

12. Senderek J, Bergmann C, Weber S, Ketelsen UP, Schorle H, et al. (2003) Mutation of the SBF2 gene, encoding a novel member of the myotubularin family, in Charcot-Marie-Tooth neuropathy type 4B2/11p15. Hum Mol Genet 12: 349-356.

13. Tersar K, Boentert M, Berger P, Bonneick S, Wessig C, et al. (2007) Mtmr13/Sbf2-Deficient Mice: An Animal Model for CMT4B2. Hum Mol Genet 16: 2991-3001.

14. Robinson FL, Dixon JE (2005) The phosphoinositide-3-phosphatase MTMR2 associates with MTMR13, a membrane-associated pseudophosphatase also mutated in type 4B Charcot-Marie-Tooth disease. J Biol Chem 280: 31699-31707.

15. Firestein R, Nagy PL, Daly M, Huie P, Conti M, et al. (2002) Male infertility, impaired spermatogenesis, and azoospermia in mice deficient for the pseudophosphatase Sbf1. J Clin Invest 109: 1165-1172.

16. Neukomm LJ, Nicot AS, Kinchen JM, Almendinger J, Pinto SM, et al. (2011) The phosphoinositide phosphatase MTM-1 regulates apoptotic cell corpse clearance through CED-5-CED-12 in C. elegans. Development 138: 2003-2014.

17. Zou W. LQ, Zhao D., Li W., Mapes J., Xie Y. and Wang X (2009) Caenorhabditis elegans Myotubularin MTM-1 negatively regulates the engulfment of apoptotic cells. PLoS Genet 5: e1000679.

18. Xue Y, Fares H, Grant B, Li Z, Rose AM, et al. (2003) Genetic analysis of the myotubularin family of phosphatases in caenorhabditis elegans. J Biol Chem 278: 34380-34386.

19. Silhankova M, Port F, Harterink M, Basler K, Korswagen HC (2010) Wnt signalling requires MTM-6 and MTM-9 myotubularin lipid-phosphatase function in Wnt-producing cells. EMBO J 29: 4094-4105.

20. Nicot AS, Toussaint A, Tosch V, Kretz C, Wallgren-Pettersson C, et al. (2007) Mutations in amphiphysin 2 (BIN1) disrupt interaction with dynamin 2 and cause autosomal recessive centronuclear myopathy. Nat Genet 39: 1134-1139.

21. Muller AJ, Baker JF, DuHadaway JB, Ge K, Farmer G, et al. (2003) Targeted disruption of the murine Bin1/Amphiphysin II gene does not disable endocytosis but results in embryonic cardiomyopathy with aberrant myofibril formation. Mol Cell Biol 23: 4295-4306.

22. Chang MY, Boulden J, Sutanto-Ward E, Duhadaway JB, Soler AP, et al. (2007) Bin1 ablation in mammary gland delays tissue remodeling and drives cancer progression. Cancer Res 67: 100-107.

23. De Camilli P, Thomas A, Cofiell R, Folli F, Lichte B, et al. (1993) The synaptic vesicle-associated protein amphiphysin is the 128-kD autoantigen of Stiff-Man syndrome with breast cancer. J Exp Med 178: 2219-2223.

24. Di Paolo G, Sankaranarayanan S, Wenk MR, Daniell L, Perucco E, et al. (2002) Decreased synaptic vesicle recycling efficiency and cognitive deficits in amphiphysin 1 knockout mice. Neuron 33: 789-804.

25. Kusumi N, Watanabe M, Yamada H, Li SA, Kashiwakura Y, et al. (2007) Implication of Amphiphysin 1 and Dynamin 2 in Tubulobulbar Complex Formation and Spermatid Release. Cell Struct Funct 32: 101-113.

26. Razzaq A, Robinson IM, McMahon HT, Skepper JN, Su Y, et al. (2001) Amphiphysin is necessary for organization of the excitation-contraction coupling machinery of muscles, but not for synaptic vesicle endocytosis in Drosophila. Genes Dev 15: 2967-2979.

27. Pant S, Sharma M, Patel K, Caplan S, Carr C, et al. (2009) AMPH-1/Amphiphysin/Bin1 functions with RME-1/Ehd1 in endocytic recycling. Nat Cell Biol 11: 1399-1410.

28. Bitoun M, Maugenre S, Jeannet PY, Lacene E, Ferrer X, et al. (2005) Mutations in dynamin 2 cause dominant centronuclear myopathy. Nat Genet 37: 1207-1209.

29. Zuchner S, Noureddine M, Kennerson M, Verhoeven K, Claeys K, et al. (2005) Mutations in the pleckstrin homology domain of dynamin 2 cause dominant intermediate Charcot-Marie-Tooth disease. Nat Genet 37: 289-294.

30. Ferguson SM, Raimondi A, Paradise S, Shen H, Mesaki K, et al. (2009) Coordinated actions of actin and BAR proteins upstream of dynamin at endocytic clathrin-coated pits. Dev Cell 17: 811-822.

31. Durieux AC, Vignaud A, Prudhon B, Viou MT, Beuvin M, et al. (2010) A centronuclear myopathy-dynamin 2 mutation impairs skeletal muscle structure and function in mice. Hum Mol Genet 19: 4820-4836.

32. Cowling BS, Toussaint A, Amoasii L, Koebel P, Ferry A, et al. (2011) Increased expression of wild-type or a centronuclear myopathy mutant of dynamin 2 in skeletal muscle of adult mice leads to structural defects and muscle weakness. Am J Pathol 178: 2224-2235.

33. Patterson EE, Minor KM, Tchernatynskaia AV, Taylor SM, Shelton GD, et al. (2008) A canine DNM1 mutation is highly associated with the syndrome of exercise-induced collapse. Nat Genet 40: 1235-1239.

34. Ferguson SM, Brasnjo G, Hayashi M, Wolfel M, Collesi C, et al. (2007) A selective activity-dependent requirement for dynamin 1 in synaptic vesicle endocytosis. Science 316: 570-574.

35. Kosaka T, Ikeda K (1983) Reversible blockage of membrane retrieval and endocytosis in the garland cell of the temperature-sensitive mutant of Drosophila melanogaster, shibirets1. J Cell Biol 97: 499-507.

36. Clark SG, Shurland DL, Meyerowitz EM, Bargmann CI, van der Bliek AM (1997) A dynamin GTPase mutation causes a rapid and reversible temperature-inducible locomotion defect in C. elegans. Proc Natl Acad Sci U S A 94: 10438-10443.
