# Supplementary material for: Defective Membrane Remodeling in Neuromuscular Diseases: Insights from Animal Models
Source: PLoS Genet. 2012 Apr 5;8(4):e1002595. doi: 10.1371/journal.pgen.1002595 (PMC3320571; doi:10.1371/journal.pgen.1002595)
Supplement: Text S1 — Glossary and useful links. (DOC) [file pgen.1002595.s003.doc]

**Glossary and useful links**

**Centronuclear myopathy**: congenital myopathies characterized by muscle weakness and mislocalization of muscle fiber nuclei.

**Charcot-Marie-Tooth neuropathies**: group of inherited peripheral neurological disorders characterized by a slowly progressive degeneration of muscles and in some forms mild loss of sensation in the limbs, fingers, and toes.

**Stiff-man syndrome**: rare neurologic disorder characterized by a progressive muscle rigidity that waxes and wanes with concurrent spasms.

**GTPase**: large class of enzymes that transform [guanosine triphosphate](http://fr.wikipedia.org/wiki/Guanosine_triphosphate) (GTP) into [guanosine diphosphate](http://fr.wikipedia.org/wiki/Guanosine_diphosphate) (GDP) and one [phosphate](http://fr.wikipedia.org/wiki/Phosphate).

**Phosphoinositides**: membrane lipids involved in membrane trafficking and signaling, playing key roles in membrane identity and protein recruitment to their site of action.

**T-tubules**: plasma membrane invaginations of the myofibers, involved in excitation-contraction coupling and calcium homeostasis in muscle.

**Schwann cells**: glia cells which plasma membrane wraps around axons in peripheral nerves and forms the myelin sheath.

**Sertoli cells:** “nurse” cell in the seminiferous tubules of the testis that nurture the developing sperm cells and consume their residual cytoplasm during spermatogenesis.

**Coelomocytes**: scavenger cells specialized in fluid phase uptake from the pseudocoelome in worms.

**For more information**

OMIM sites for the discussed diseases:

CNM:

ADCNM: <http://omim.org/entry/160150>

ARCNM: <http://omim.org/entry/255200>

XLCNM: <http://omim.org/entry/310400>

CMT:

CMT4B1: <http://omim.org/entry/601382>

CMT4B2: <http://omim.org/entry/604563>

CMTDIB: <http://omim.org/entry/606482>

http://www.molgen.ua.ac.be/CMTMutations/Mutations/Default.cfm

Stiff-man:

<http://omim.org/entry/184850>

Dynamin and amphiphysin homepages:

<http://www2.mrc-lmb.cam.ac.uk/groups/hmm/Dynamin/>

<http://www2.mrc-lmb.cam.ac.uk/groups/hmm/Amphiphysin/amphiphysin.html>
